# Genetic polymorphism of *Plasmodium falciparum* circumsporozoite protein in Kigali, Rwanda

**DOI:** 10.3389/fpara.2025.1679131

**Published:** 2025-11-04

**Authors:** Sandra Noukimi Fankem, Jean-Bosco Mbonimpa, Edgar Mutebwa Kalimba, Mariama Telly Diallo, Jacob Souopgui

**Affiliations:** ^1^ Laboratory of Embryology and Biotechnology, Department of Molecular Biology, Faculty of Science, Université Libre de Bruxelles, Gosselies, Belgium; ^2^ Rwanda Malaria Research Lab, King Faisal Hospital Rwanda, Kigali, Rwanda

**Keywords:** circumsporozoite protein (CSP), *Plasmodium falciparum*, malaria vaccine, genetic diversity, Kigali

## Abstract

**Introduction:**

Malaria remains a major public health challenge across sub-Saharan Africa, with Plasmodium falciparum responsible for the vast majority of cases and deaths. In Rwanda, although control measures have led to significant progress, malaria continues to be endemic, with urban centers like Kigali experiencing continuous transmission. With the recent rollout of malaria vaccines such as RTS,S and R21, understanding the genetic variability of vaccine-targeted antigens is essential for anticipating and enhancing vaccine performance.

**Methods:**

This study investigated the genetic diversity of the Plasmodium falciparum circumsporozoite protein (Pfcsp) gene among 245 clinical isolates collected between October 2021 and June 2023 at King Faisal Hospital, a referral center in Kigali, Rwanda. PCR amplification of the csp locus was performed, and the resulting amplicons were sequenced using Oxford Nanopore Technology (ONT) employing the R10.4 flow cell chemistry, allowing for high-resolution haplotype reconstruction and detection of polymorphic sites across the gene.

**Results:**

A total of 48 distinct haplotypes were identified, indicating high haplotype diversity (Hd = 0.8899) but moderate nucleotide diversity (p = 0.00834), suggesting immune-driven balancing selection. The N-terminal region was highly conserved across isolates, including full conservation of the KLKQP motif, reinforcing its functional importance in hepatocyte invasion. In contrast, the central repeat region exhibited substantial variability in NANP/ NVNP tetrapeptide repeat numbers, and the C-terminal region, particularly the Th2R and Th3R epitopes showed extensive polymorphism. Notably, fewer than 1% of sequences matched the 3D7 vaccine strain, and several key amino acid positions associated with vaccine escape showed high mutation frequencies.

**Discussion:**

Our findings suggest that the genetic divergence of circulating csp variants in Kigali could be a factor influencing vaccine performance, underscoring the importance of ongoing molecular surveillance to guide eventual vaccine implementation in Rwanda and other endemic regions

## Introduction

Malaria remains a major global health concern, disproportionately affecting sub-Saharan Africa, which accounts for over 90% of the disease’s burden in terms of cases and mortality (WHO, 2024). According to the World Health Organization (WHO), approximately 263 million malaria cases and 597,000 associated deaths were reported globally in 2023, an increase of over 11 million cases compared to the previous year (WHO, 2024). This increase trend, despite the ongoing implementation of control strategies, highlights the urgent need for more robust and complementary interventions such as effective vaccines alongside traditional tools like chemoprevention and vector control (Bhatt et al., 2015; RTS,S Clinical Trials Partnership, 2015).

Rwanda has made remarkable progress in malaria control, significantly reducing disease incidence and mortality over the past two decades (Umugwaneza et al., 2025). However, sustained human movement between Kigali and malaria-endemic regions presents a risk of parasite reintroduction, which may lead to local outbreaks as it has been observed between 2024 and 2025 (Mbabazi, 2025). This outbreak has pushed the Rwandan government to add dihydroartemisinin-piperaquine and artesunate-pyronaridine in the national malaria treatment guidelines as the second-line for treatment of uncomplicated malaria case (Mbabazi, 2025). To help strengthen malaria control strategies, the introduction of a vaccine would be important, and prior to its implementation, molecular surveillance of vaccine targets should be conducted.

In recent years, WHO has approved two malaria vaccines targeting *Plasmodium falciparum* sporozoites. The RTS,S/AS01 vaccine received full recommendation in 2021, followed by the R21/Matrix-M vaccine in 2023 for use in children residing in areas with moderate to high transmission (Adepoju, 2023; RTS,S Clinical Trials Partnership, 2015). Both vaccines are designed against the *P. falciparum* circumsporozoite protein (*Pfcsp*), a surface antigen critical for liver-stage invasion following mosquito transmission (Collins et al., 2017; Laurens, 2019). RTS,S/AS01 comprises the central NANP repeat and the C-terminal domain of PfCSP protein, fused with the hepatitis B surface antigen and formulated with the AS01 adjuvant (Al-obeidee and Al-obeidee, 2024). R21/Matrix-M incorporates a similar antigenic region but uses a more balanced ratio of PfCSP to HBsAg, which has been associated with enhanced immunogenicity (Al-obeidee and Al-obeidee, 2024).

Although these vaccines represent major advances in malaria prevention, their protective efficacy is partly influenced by the genetic variability of the PfCSP antigen in circulating parasite populations. The PfCSP protein consists of three regions: a relatively conserved N-terminal domain, a central repeat region composed predominantly of NANP and NVDP motifs, and a polymorphic C-terminal region that contains critical T-cell epitopes, specifically the Th2R and Th3R regions recognized by CD4 + and CD8 + T cells, respectively (Al-obeidee and Al-obeidee, 2024; Laurens, 2019).

Numerous molecular epidemiological studies across Africa have documented substantial regional variation in PfCSP haplotypes, often showing that the 3D7 vaccine-type allele is underrepresented, present in as few as 5–10% of local parasite populations in certain settings (Huang et al., 2020; Maina et al., 2024). An analysis of the RTS,S Phase III trial highlighted the importance of this diversity by demonstrating that vaccine efficacy was significantly higher (50.3%) against parasites matching the 3D7 vaccine allele, compared to 33.4% for infections caused by non-matching strains (Neafsey et al., 2015). These findings underscore the relevance of characterizing local *csp* diversity to anticipate allele-specific immune responses and guide vaccine introduction policies.

Despite Rwanda’s ongoing efforts to explore malaria vaccine deployment, there is currently no published data on the genetic diversity of the *csp* gene in local *P. falciparum* populations. Given Kigali’s central role in national and regional movement and transmission dynamics, genomic surveillance in this urban setting is both timely and necessary. A deeper understanding of *csp* polymorphisms, particularly within immunologically relevant domains, will provide baseline data essential for evaluating vaccine coverage and guiding strategic implementation.

This study aimed at characterizing the sequence diversity of the *csp* gene in *P. falciparum* isolates collected from patients in Kigali, Rwanda. Using Oxford Nanopore Technology, we assessed the extent of polymorphism in the Th2R and Th3R epitope regions and explore haplotype structure and selection pressures.

## Methods

### Sample collection, DNA extraction and sequencing

Samples collected for this study was part of a global cross-sectional study performed between October 2021 and June 2023. A total of 310 samples was selected from leftover blood samples originally obtained for routine malaria diagnosis of patients attending King Faisal Hospital Rwanda in Kigali. Sample collection was distributed unevenly across the study period: 38 samples were collected between October–December 2021, 216 samples between January–December 2022, and 56 samples between January–June 2023. Due to this imbalance and the cross-sectional nature of the design, all samples were pooled for analysis to provide an overall assessment of haplotype diversity in Kigali during the study period.

The collected samples were subsequently preserved by mixing with DNA/RNA Shield (Zymo Research), following the manufacturer’s instructions. Samples were stored at −20°C and later transported to Belgium for further molecular analysis. Total genomic DNA was extracted using Maxwell^®^ RSC Whole Blood DNA Kit (Promega – AS1520). A total of five *Plasmodium falciparum* genes, namely *crt, mdr1, dhfr, dhps*, and *csp* were amplified using a multiplex PCR approach as previously described (Girgis et al., 2023). PCR products were used for library preparation employing Ligation sequencing amplicons - Native Barcoding Kit 24 V14 (SQK-NBD114.24) from Oxford Nanopore Technology (ONT, UK). Prepared library was run on Flongle Flow Cells (R10.4.1) for 24hrs using MinKNOW software version 24.06.16 (ONT, UK).

### Bioinformatic analysis

Basecalling and demultiplexing of reads were conducted using Guppy version 7.0.9, within the MinKNOW v23.07.5 software. The process was configured with a minimum barcode quality threshold of 60 and a minimum read quality score of 9. The resulting reads were aligned to the *Plasmodium falciparum* 3D7 reference genome using Minimap2 (version 2.28-r1209) (Li, 2021), producing output in SAM format. Samtools version 1.21 (Danecek et al., 2021) was used to convert SAM to BAM file and reads mapping to *csp* gene were extracted.

Coverage across the *csp* amplicon was calculated using Samtools depth, and metrics including minimum, maximum, mean, and median coverage were summarized. Samples were considered to have passed quality control if they met the following criteria: minimum average read depth of 50X and at least 80% of the amplicon covered at ≥ 50X. Samples failing any of these criteria were excluded.

Reads mapping to the 3D7 csp reference sequence were extracted and used as input to generate consensus using Amplicon_sorter 83 (Vierstraete and Braeckman, 2022) with a similarity cut-off of 96%. The consensus sequences obtained were trimmed to include only the portion of the sequence within the *csp* primer pair used. The resulting consensus sequences were then mapped against the 3D7 reference sequence using the Clustal Omega tool in Ugene version 45.0. To investigate the genetic distance between Kigali *csp* sequences and the 3D7 strains, a genetic distance tree was generated using the maximum likelihood method in MEGA7 software (Kumar et al., 2016). Genetic diversity indices, including the number of haplotypes (H), haplotype diversity (Hd), and the number of segregating sites (S), were estimated with DnaSP version 6.12.03 (Rozas et al., 2017). Additionally, multiple sequence alignment of the amino acid sequence of haplotypes was performed using Ugene software version 45.0 and haplotype frequencies oh TH2R and TH3R was computed with GraphPad Prims version 9.0.0.

## Results

A total of 310 blood samples were collected between October 2021 and June 2023. Participants were predominantly male (60.3%), and most were aged 21–50 years (48.1%). Other age groups included 6–20 years (23.9%), 0–5 years (15.8%), and over 50 years (12.3%). Parasitemia levels were mostly low to moderate, with 41.0% of individuals showing <1,000 parasites/µL, 41.6% between 1,000–10,000 parasites/µL, and 17.4% exceeding 10,000 parasites/µL. Out of the 310 samples selected at the King Faisal Hospital Rwanda, 245 were successfully amplified, sequenced and passed quality filtering steps, allowing for the generation of consensus sequences for *csp* amplicons. Among retained samples, sequencing depth ranged from 50X to 7,831X, with a mean of 1,710.3X and a median of 1,073X. Mono-infections accounted for 93.5% of the isolates, with the remaining samples representing multiple infections. Sequencing analysis of genetic diversity indices using DnaSP showed that the full-length of examined isolates of *csp* sequences comprised 48 haplotypes with a diversity of 0.8899, nucleotide diversity of 0.00834 and 63 segregating sites. The 48 identified haplotypes comprised of 27 haplotype singletons (having only a single sequence) and 21 haplotypes with more than 2 sequences. Sequencing analysis excluding singleton haplotypes revealed the presence of 27 haplotypes with a diversity of 0.8560, nucleotide diversity of 0.00812 and 58 segregating sites. These results did not substantially alter haplotype diversity indices or variant distributions (P = 0.125, Wilcoxon test), indicating that our results are robust to the treatment of singletons. Therefore, focus was given to the 21 haplotypes (218 out of 245 isolates, 88.97%) having more than 2 sequences.

### Genetic diversity in the N-terminal region of *csp* gene

**Figure 1:**
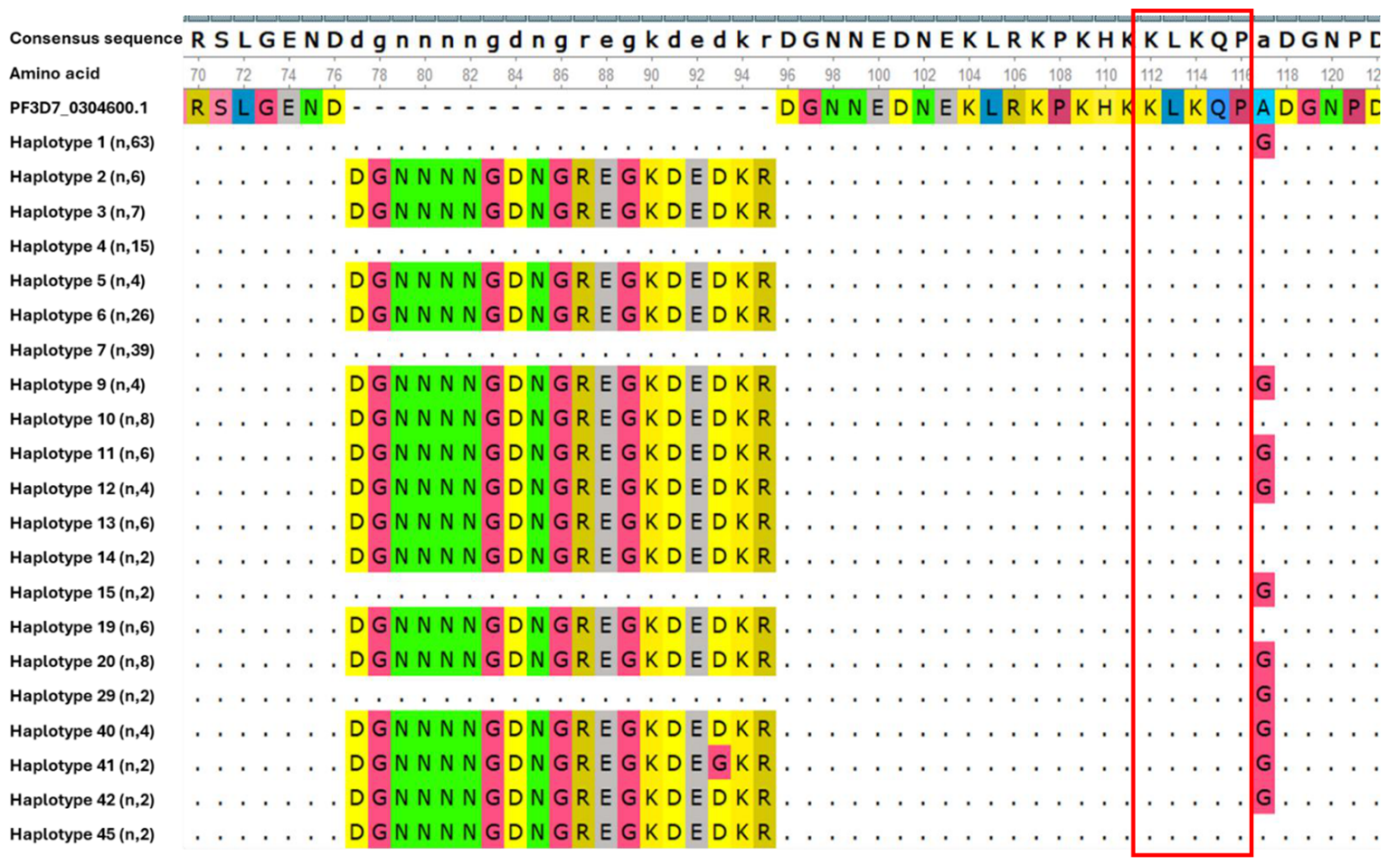
Genetic diversity in N-terminal region of csp gene in isolates circulating in Kigali. Each haplotype represents all isolates within that group, with the number of isolates per haplotype indicated in parentheses. Dash (-) indicate the insertion of the 19 amino acid segments and dots (.) indicate identical amino acid residues to those in the reference sequence.

The genetic polymorphism within the N-terminal region of the *csp* gene was evaluated using the reference sequence (PF3D7_0304600.1). We observed that this region was highly conserved (121 out of 218 isolates, 55.5%) among all samples analyzed. Three distinct haplotypes groups were identified. Group 1 (haplotypes 2, 3, 6, 9, 10, 11, 12, 13, 14, 19, 20, 40, 42 and 45) featured an insertion of a 19 amino acid segment at position 77 (DGNNNNGDNGREGKDEDKR), group 2 (haplotype 41) an insertion of DGNNNNGDNGREGKDE**G**KR at position 77, while group 3 (haplotypes 1, 4, 7, 15 and 29) lacked this insertion. In addition to the insertion, 44.5% of isolates had the A98G single nucleotide polymorphism (**Figure 1**). However, no mutation was observed in the KLKQP motif, which plays a role in parasite invasion, and which was maintained across all isolates.

### Genetic diversity in the central repeat region of *csp* gene

**Figure 2:**
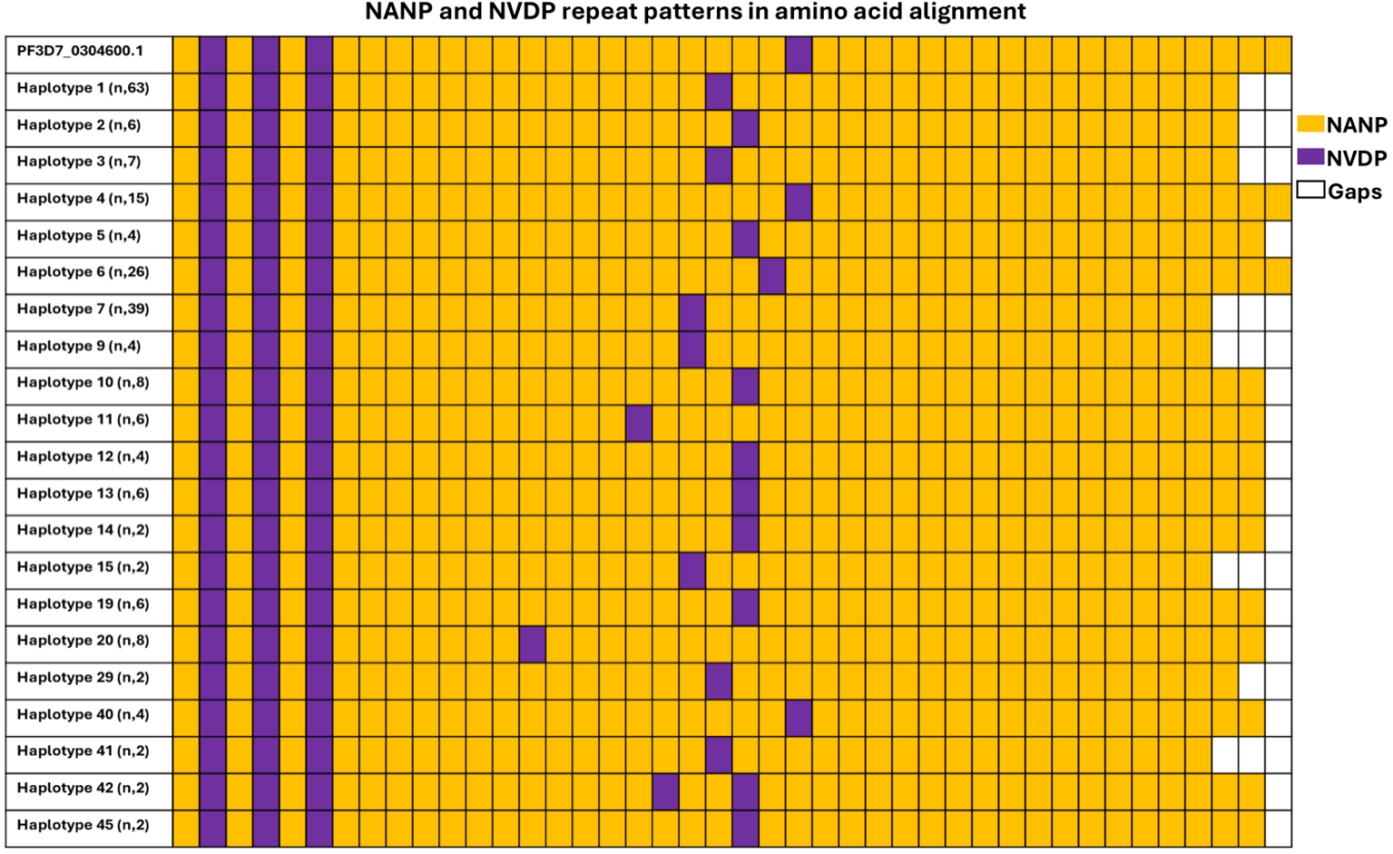
Genetic diversity in central region of csp gene among Kigali isolates. Central repeat region in Kigali isolates was compared to the reference sequence 3D7 (PF3D7_0304600.1). Color code represents tetra-peptide motifs, orange represents NANP, and purple represents NVDP.

Within the central repeat region, the NANP region remained relatively conserved across isolates circulating in Kigali. The number of NANP/NVDP repeats were analyzed and compared among Kigali haplotypes and the reference gene as shown in **Figure 2**. In the 3D7 reference *csp* sequence, the number of NANP and NVDP motifs were 38 and 4 respectively. In Kigali isolates, the number of NANP repeats present ranged from 35 to 38, with 36 being the most frequent (84/218, 38.53%) and, the NVDP repeats ranged from 4 to 5, contributing to the polymorphism in the central repeat region. Finally, the total number of NANP and NVDP repeats per haplotype varied from 39 to 43, where the majority of isolate contained 39 of NANP and NVDP repeats.

### Genetic diversity in the C-terminal region of *csp* gene

**Figure 3:**
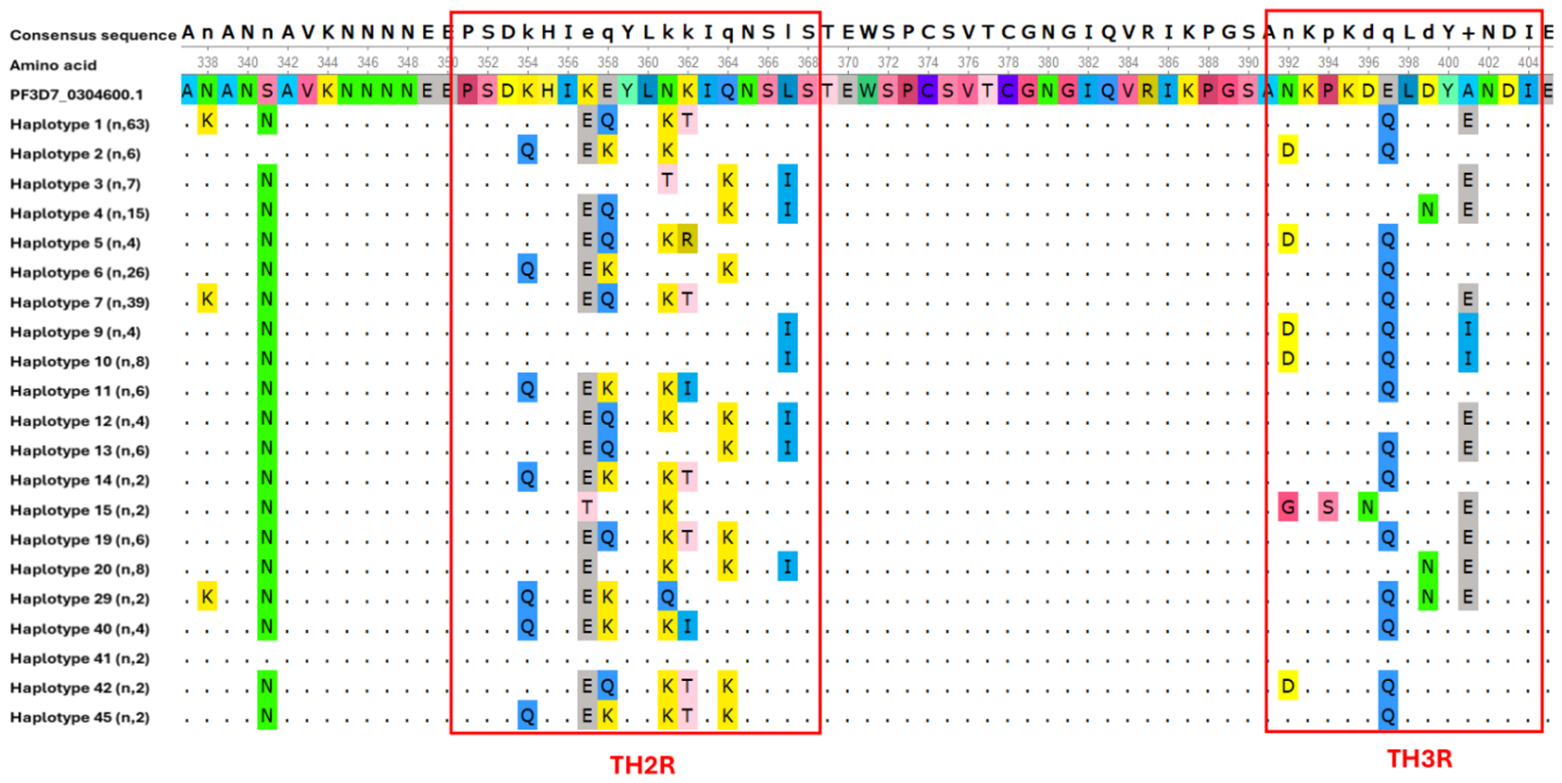
Polymorphism in the C-terminal region of csp gene among Kigali isolates. Multiple sequence alignment of 21 haplotypes circulating in Kigali. Dots (.) indicate identical amino acid residues to those in the reference sequence.

**Figure 4:**
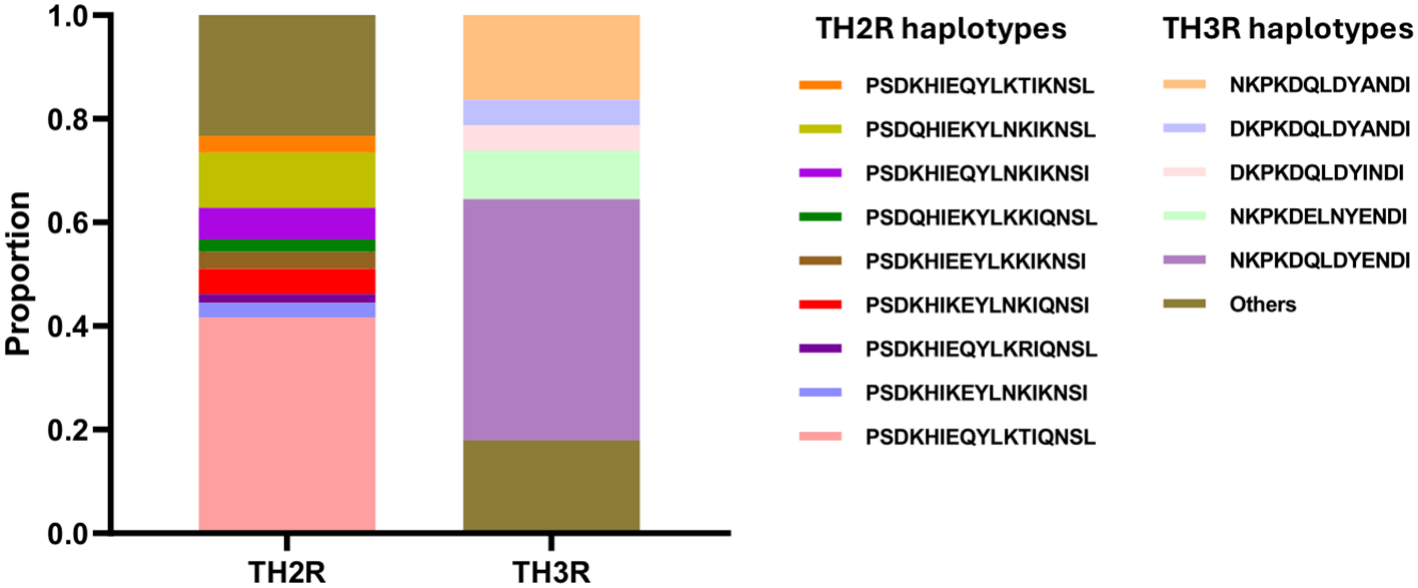
Haplotypes proportions in C-terminal region of csp gene. TH2R and TH3R haplotype proportions in 245 isolates circulating in Kigali. Amino acids in TH2R haplotypes range from 311 to 327, and in TH3R from 352 to 364.

The C-terminal region showed higher levels of single nucleotide polymorphism compared to the other regions. These polymorphisms were principally observed in the Th2R and Th3R epitopes. Multiple non-synonymous mutations were detected within these epitopes relative to the 3D7 reference sequence used in the RTS,S and R21/Matrix-M vaccines. Unexpectedly, our analysis revealed that only one *csp* haplotype (haplotype 41) was identical to the vaccine strain in both the Th2R (^311^PSDKHIKEYLNKIQNSL^327^) and Th3R (^352^NKPKDELDYANDI^364^) epitope regions (**Figure 3**). A total of 21 single nucleotide polymorphism was identified in C-terminal region. All other haplotypes displayed at least one amino acid substitution compared to the reference strain, emphasizing the high level of polymorphism among the identified variants. Detailed analysis of the Th2R region revealed over 10 distinct haplotypes, with PSDKHIEQYLKTIQNSL (102/245, 41.63%) and PSDQHIEKYLNKIKNSL (26/245, 10.61%) being the most prevalent. In contrast, fewer haplotypes were observed in the Th3R region, where NKPKDQLDYENDI (114/245, 46.53%) and DKPKDQLDYINDI (40/245, 16.32%) emerged as the dominant variants (**Figure 4**).

### Genetic distance analysis of *csp* isolates in Kigali

**Figure 5:**
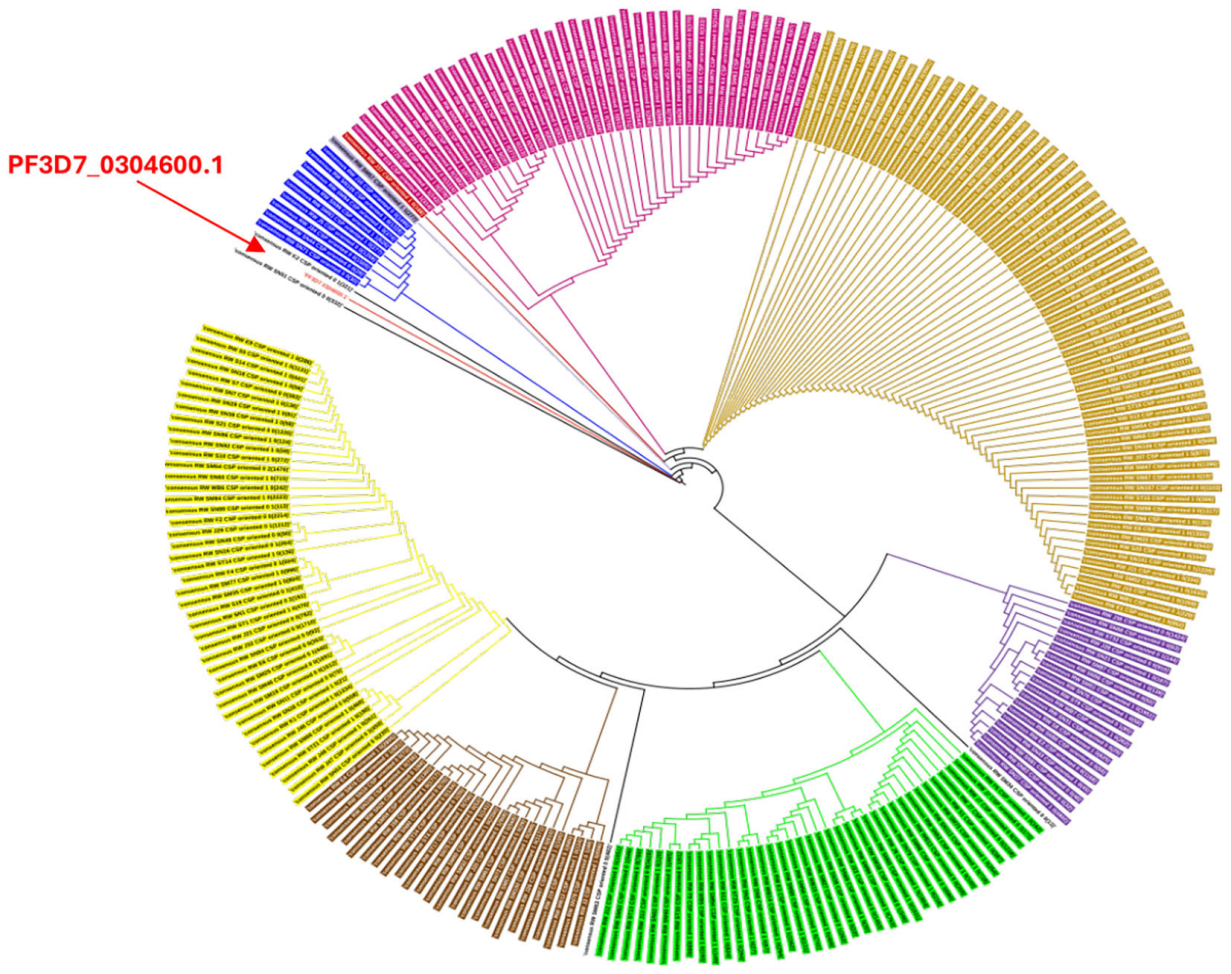
Genetic tree of csp isolates in Kigali. The color coding in the genetic tree indicates isolates with similar characteristics in the csp gene.

The genetic tree (**Figure 5**) of *csp* isolates circulating in Kigali showed a high degree of diversity with the reference sequence being genetically distinct from most of the haplotypes. We observed the presence of 3 main clusters that represent the dominant haplotypes circulation in the study population.

## Discussion

Our study aimed at understanding the genetic architecture of *csp* isolates circulating in the city of Kigali in Rwanda. However, samples were collected only from patients attending King Faisal Hospital, a referral hospital. A potential limitation of our findings is the influence of urban referral bias on haplotype distribution. Referral hospitals typically admit patients with more severe or treatment-refractory infections, which may enrich the sample for resistant haplotypes while underrepresenting asymptomatic or mild infections circulating in the community. Moreover, referral centers often draw patients from peri-urban or high-transmission catchment areas, which may not reflect broader population dynamics. As a result, haplotype frequencies observed in urban referral settings could overestimate the prevalence of resistance-associated alleles compared with the general population. Future surveillance efforts should integrate samples from community and peripheral health facilities to mitigate referral bias and generate a more representative picture of haplotype dynamics.

Analysis of these isolates revealed a total of 48 distinct haplotypes, with a haplotype diversity of 0.8899, nucleotide diversity (π) of 0.00834, and 63 segregating sites. The high haplotype diversity indicates substantial genetic variation within the local *P. falciparum* population, suggesting that multiple *csp* variants are co-circulating in Kigali. This is comparable to findings from other East African regions such as Kenya, where 109 *csp* haplotypes were reported, with each infection harboring novel, recurrent, or persistent haplotypes (Sumner et al., 2021). Despite this, the moderate nucleotide diversity and the presence of 27 singleton haplotypes suggest that many haplotypes differ by only a few nucleotide changes. This pattern of high haplotype but moderate nucleotide diversity is consistent with balancing selection acting on immunogenic regions, where selective pressure maintains multiple variants without excessive divergence. Investigating the genetic diversity of *P. falciparum* in Kigali, Rwanda, is a key to generate data that support the future rollout of malaria vaccine in the country.

The N-terminal region of the *csp* gene is highly conserved, displaying low genetic polymorphism with KLKQP motif uniformly conserved across all the analyzed Kigali isolate. The KLKQP motif plays an important role during sporozoite invasion of the hepatocytes (Rathore et al., 2002). These results are consistent with findings from other malaria-endemic regions such as Kenya, Ethiopia and Ghana (Amegashie et al., 2020; Maina et al., 2024; Mandefro et al., 2025). However, 44.5% of sequences carried a non-synonymous A98G substitution, and most exhibited a 19-amino-acid insertion within the central portion of the N-terminal region. These findings align with previous reports from other settings (Mandefro et al., 2025; Mohamed et al., 2021). The N-terminal domain plays a critical role in sporozoite invasion of hepatocytes (Dundas et al., 2019; Rathore et al., 2002), and the presence of non-synonymous mutations and insertions may reflect adaptive mechanisms by the parasite to escape host immune pressure (Rathore et al., 2002).

The central repeat region of the *csp gene* is an immunodominant epitope and a key component of the RTS,S malaria vaccine (Gordon et al., 1995). Variation in the number of tetrapeptide repeats is a major contributor to the genetic polymorphism observed in the *csp* gene. This study identified variability in the number of these tetrapeptide repeats among isolates, with most samples exhibiting between 39 and 43 repeats. Although some researchers argue that variations in repeat number do not significantly influence RTS,S vaccine efficacy (Neafsey et al., 2015), others have suggested a potential link between repeat number and the structural stability of the CSP protein (Escalante et al., 2002). Nonetheless, the functional consequences and immunological impact of this variation remain poorly understood. Given the widespread nature of this polymorphism, further in-depth investigations into the repeat region are warranted to better elucidate its role in vaccine performance and parasite biology.

Analysis of the C-terminal region of the *csp* gene revealed substantial polymorphism, particularly within the Th2R and Th3R epitopes, which are recognized by CD4^+^ and CD8^+^ T cells, respectively. In the isolates analyzed in this study, only 2% matched the C-terminal region of 3D7 strain sequence used in the RTS,S and R21 vaccines. This low prevalence of 3D7-matching haplotypes is consistent with findings from other African regions, where such matches have been reported in fewer than 5% of circulating parasite strains (Huang et al., 2020; Mandefro et al., 2025). Previous studies examining the genetic diversity of *csp* and its impact on RTS,S/AS01 vaccine efficacy have suggested that mismatches, especially at specific amino acid positions such as 299, 301, 317, 354, 356, 359, and 361, may reduce vaccine effectiveness (Neafsey et al., 2015). In our analysis, several of these positions showed high levels of variation: S301N was present in 96.3% of sequences, E317K and E317Q in 85.8%, and A361E or A361I in 75.2%. The extremely low frequency of vaccine-matching haplotypes (<1%) among Kigali isolates may negatively influence vaccine performance, as prior evidence indicates that RTS,S efficacy is higher against haplotypes matching the vaccine strain (Neafsey et al., 2015). However, the precise mechanisms by which these mutations impact vaccine-induced immunity remain unclear.

Further analysis of *csp* sequences confirmed extensive genetic diversity with distinct clade structures. The 3D7 reference strain, used in current vaccines, clustered within a minor lineage, underscoring its limited representation among circulating haplotypes. This divergence highlights the potential for allele-specific immune evasion and supports the need for ongoing molecular surveillance to guide vaccine deployment strategies.

In summary, this study provides a comprehensive analysis of the genetic diversity of *P. falciparum csp* gene in isolates collected from Kigali, Rwanda. The N-terminal region of *csp* was highly conserved across isolates, particularly the KLKQP motif, which supports its critical role in hepatocyte invasion. In contrast, the central repeat and C-terminal regions showed considerable variability. Variation in NANP/NVNP repeat numbers and high polymorphism within the Th2R and Th3R epitopes, particularly at vaccine-relevant amino acid sites underscore the challenges posed by genetic mismatches to the efficacy of current vaccines like RTS,S. However, the implications of our findings for the R21 vaccine warrant careful consideration. Although R21 and RTS,S both target the circumsporozoite protein (CSP), R21 differs in formulation and immunogenicity, with evidence of higher antibody titers and potentially greater durability of response. Whether the haplotype distributions we report will influence R21 efficacy in a manner similar to, or divergent from, RTS,S remains uncertain. It is reasonable to hypothesize that R21 may demonstrate higher efficacy; however, parasite genetic variation in CSP must be evaluated for both vaccines, as differential immune pressure could yield distinct patterns of protection. Comprehensive monitoring of haplotype distribution alongside vaccine performance will be essential to generate robust, data-driven conclusions in the Rwandan context.

## Data Availability

The data presented in the study are deposited in the European Nucleotide Archives (ENA) repository, accession number PRJEB101030 (ERP182457).
